# Comparative effectiveness of massage combined with lifestyle intervention and lifestyle intervention alone for simple obesity: A systematic review and meta-analysis

**DOI:** 10.1097/MD.0000000000041074

**Published:** 2025-01-10

**Authors:** Gaofeng Wang, Hongyu Ju, Zepeng Zhang, Xingquan Wu, Heli Niu, Lili Zhang, Lili Chen, Huijuan Lou, Yonggang Yang

**Affiliations:** a Department of Traditional Chinese Medicine, Baicheng Medical College, Baicheng, Jilin Province, China; b Department of Traditional Chinese Medicine, the Affiliated Hospital of Baicheng Medical College, Baicheng, Jilin Province, China; c Department of Endocrine and Metabolic Diseases, Baicheng Municipal Hospital, Baicheng, Jilin Province, China; d Department of Lab Center, the Affiliated Hospital of Changchun University of Chinese Medicine, Changchun, Jilin Province, China; e Department of Tuina, the Affiliated Hospital of Changchun University of Chinese Medicine, Changchun, Jilin Province, China.

**Keywords:** lifestyle intervention, massage, meta-analysis, obesity

## Abstract

**Background:**

This study aimed to assess the comparative effectiveness of massage combined with lifestyle intervention and lifestyle intervention alone in patients with simple obesity.

**Methods:**

The PubMed, Embase, Cochrane Library, CNKI, VIP Database, and Wanfang Data were searched. Meta-analysis was conducted in accordance with the 2020 Preferred Reporting Items for Systematic Reviews and Meta-Analysis guidelines. Primary outcomes were body weight (BW) and body mass index (BMI). Secondary outcomes were waist circumference (WC), hip circumference (HC), total cholesterol (TC), triglyceride (TG), low-density lipoprotein cholesterol (LDL-C), high-density lipoprotein cholesterol (HDL-C), fasting insulin (FINS), and homeostasis model assessment–insulin resistance (HOMA-IR) and adverse events.

**Results:**

Thirteen randomized controlled trials were included. The meta-analysis showed that massage combined with lifestyle intervention significantly decreased BW (mean difference [MD]: −4.85; 95% confidence interval [CI]: −8.25 to −1.46; *P* = .005), BMI (MD: −2.65; 95% CI: −4.05 to −1.24; *P* = .0002), WC (MD: −3.63; 95% CI: −6.28 to −0.98; *P* = .007), TC (MD: −0.52; 95% CI: −0.84 to −0.20; *P* = .001), TG (MD: −0.23; 95% CI: −0.45 to −0.02; *P* = .003), LDL-C (MD: −0.48; 95% CI: −0.54 to −0.42; *P* < .00001), HDL-C (MD: −0.11; 95% CI: −0.17 to −0.05; *P* = .0004), FINS (MD: −1.64; 95% CI: −3.16 to −0.12; *P* = .03), and HOMA-IR (MD: −0.42; 95% CI: −0.65 to −0.18; *P* = .0005) compared with lifestyle intervention alone. In subgroup analyses, more obvious reduction in BMI (*P* = .02, *I*^2^ = 80.3%) for the children and adolescents subgroup, more obvious reduction in HC (*P* = .04, *I*^2^ = 76.1%) for the adults subgroup, more significant reduction in TC (*P* < .00001, *I*^2^ = 98.3%), LDL-C (*P* < .00001, *I*^2^ = 95.6%), and HDL-C (*P* < .0001, *I*^2^ = 94.1%) for intermittent treatment subgroup and more significant reduction in TC (*P* < .00001, *I*^2^ = 95.9%) and HDL-C (*P* < .0001, *I*^2^ = 94.1%) for treatment times ≤30 subgroup were detected.

**Conclusions:**

Compared with lifestyle intervention alone, massage combined with lifestyle intervention significantly decreased BW, BMI, WC, TC, TG, LDL-C, FINS, and HOMA-IR, but produced less effect in increasing HDL-C. And different ages, treatment intervals, and treatment times can all affect treatment outcomes.

## 1. Introduction

Obesity refers to the state of excessive or abnormal body fat accumulation.^[1]^ Simple obesity is the presence of obesity without other diseases, often caused by unhealthy lifestyles, including poor dietary habits and limited physical activity.^[2]^ And, obesity has been regarded as 1 of the main risk factors for noncommunicable disorders, including cardiovascular diseases, diabetes, certain cancers, and disability.^[3–5]^ The prevalence of obesity has steadily increased, making obesity a health concern worldwide.^[6,7]^ According to the World Health Organization, in 2020, there were 1.9 billion overweight adults and 0.6 million obese adults.^[8]^ Obesity has emerged as a leading health problem over the past century.^[9]^

The main treatment options with sufficient evidence-based support for obesity are lifestyle intervention, pharmacotherapy, and bariatric surgery.^[9]^ Lifestyle interventions aimed at changing dietary behaviors and physical activity are usually the first choice for weight loss.^[10,11]^ However, management of obesity through behavioral changes aiming at cutting down caloric intake and increasing physical activity is frequently unsuccessful,^[12]^ making weight loss a goal that is very often difficult to achieve.^[13,14]^ Thus, patients with obesity have begun seeking alternative forms of health care for weight loss.^[15,16]^ Massage is such an appealing alternative to the increasingly obesity population.^[15,17]^ And, massage has been widely used in the field of body contouring and weight loss in China, France, the United States, and many other nations.^[18–20]^

The effectiveness of massage on obesity is gradually being reported. Several early studies have shown improvements in body contour and skin texture after massage treatment.^[18,19,21,22]^ Foster et al^[23]^ have shown reductions in thigh and infraumbilical circumference following treatment with massage. A study conducted by Brightman et al^[24]^ has demonstrated improvement in arm and postpartum abdominal and flank subcutaneous fat deposits and skin laxity after massage. Boey et al^[25]^ have demonstrated that massage treatment can enhance the clinical outcome of cryolipolysis treatment. Lee et al^[26]^ have shown a significant reduction in weight, waist circumference (WC), body mass index (BMI), and serum lipids level after massage treatment. Klaudia Antoniak et al^[27]^ found that massage can make a favorable change in lipid profile in obese people. Nozomi Donoyama et al^[28]^ observed that adiponectin levels in blood increased in mildly obese women after massage treatment. However, current evidence supporting massage for obesity is still largely limited. An early systematic review even advocated acupuncture/massage had no effect on body weight (BW).^[29]^ Yet the review did not separate the effectiveness of massage on obesity from that of acupuncture and has not been generally accepted for not well-performed clinical trials.^[29]^ Widespread use of massage for the treatment of obesity has been accompanied by a growing number of reports discussing the effects of massage on weight loss. It is important to objectively evaluate and analyze the efficacy of massage in obesity treatment. However, there are no systematic reviews and meta-analyses that focus on the treatment effectiveness of massage for patients with simple obesity. As an alternative method, massage therapy was usually conducted combined with lifestyle intervention for weight loss. Therefore, this systematic review and meta-analysis aimed to compare the clinical efficacy of massage combined with lifestyle intervention and lifestyle intervention alone for patients with obesity using randomized controlled trials (RCTs).

## 2. Materials and methods

### 2.1. Study registration

This systematic review and meta-analysis was registered in the International Prospective Register of Systematic Reviews database (CRD42022370830), and conducted in accordance with the 2020 Preferred Reporting Items for Systematic Reviews and Meta-Analysis Statement guidelines.^[30]^

### 2.2. Data sources

Three English-language databases (PubMed, Embase, and Cochrane Library) and 3 Chinese-language databases (China National Knowledge Infrastructure, VIP Database, and Wanfang Data) were systematically searched for eligible studies from inception to December 11, 2022. The publication language was restricted to Chinese and English. The search strategy consisted of the following 3 components: clinical condition (obesity, overweight), 1 intervention method (diet, meals, exercise, lifestyle, behavioral education), and another intervention method (massage, anmo, tuina, acupressure, manipulate*). The complete search strategy for PubMed is displayed in Table 1, and it was modified as necessary for other databases. In addition, the references of the published papers were also manually searched (in Chinese and English) to identify cited articles that had not been found in the electronic search.

**Table 1:** Search strategy for the PubMed database.

Number	Search terms
#1	Massage (MeSH)
#2	Anmo (MeSH)
#3	Tuina (MeSH)
#4	Acupressure (MeSH)
#5	Manipulate* (MeSH)
#6	Massage (ti, ab)
#7	Anmo (ti, ab)
#8	Tuina (ti, ab)
#9	Acupressure (ti, ab)
#10	Manipulate* (ti, ab)
#11	#1 or #2 or #3 or #4 or #5 or #6 or #7 or #8 or #9 or #10
#12	Diet (MeSH)
#13	Meals (MeSH)
#14	Exercise (MeSH)
#15	Life style (MeSH)
#16	Behavioral education (MeSH)
#17	Diet (ti, ab)
#18	Meals (ti, ab)
#19	Exercise (ti, ab)
#20	Life style (ti, ab)
#21	Lifestyle (ti, ab)
#22	Behavioral education (ti, ab)
#23	#12 or #13 or #14 or #15 or #16 or #17 or #18 or #19 or #20 or #21 or #22
#24	Overweight (MeSH)
#25	Obesity (MeSH)
#26	Overweight (ti, ab)
#27	Obesity (ti, ab)
#28	#24 or #25 or #26 or #27
#29	#11 and #23 and #28

ti, ab = title/abstract.

### 2.3. Eligibility criteria

RCTs that compared the effectiveness of massage combined with lifestyle intervention and lifestyle intervention alone for patients with simple obesity were considered eligible in our meta-analysis.

Studies were included if they met the following criteria. (1) The subjects were definitely classified as having simple obesity. (2) Eligible interventions were massage combined with lifestyle intervention, where lifestyle interventions involved diet, exercise, behavioral education, or a combination of these methods. The comparison group was lifestyle intervention without additional method. (3) Study design: RCT design. (4) Outcomes: BW, BMI, WC, hip circumference (HC), blood serum lipid level, blood glucose, insulin sensitivity, and adverse events.

The exclusion criteria were as follows: (1) studies with unclear diagnosis and treatment standards; (2) studies with subjects diagnosed as secondary obesity, such as drug-induced obesity, hypothalamic syndrome, pituitary adenoma, hypercortisolism, polycystic ovary syndrome, postpartum obesity; (3) studies with traditional Chinese medicine or western medicine as intervention means, and studies with nonmassage treatment as intervention means; (4) nonclinical research (animal experiment, meta-analysis, review, guideline, protocol, published abstracts, comments, or letters), personal experience, conference papers, and mechanism research studies with non-effect evaluation; and (5) studies that have been published twice or have similar research data.

### 2.4. Data extraction

Two reviewers (Lili Chen and Heli Niu) independently screened the studies using the software EndNote X9 and extracted the data of the included trials with a structured data extraction form by Microsoft Excel 2018 with the following information: first author’s name, publication year, country, language, sample size, mean age, male proportion, intervention and control, treatment duration, and reported outcomes. A third author (Huijuan Lou) verified these data.

### 2.5. Quality assessment

Two reviewers (Lili Zhang and Zepeng Zhang) independently assessed the quality of each trial according to the Cochrane Risk of Bias (RoB) 2.0 tool, which contained 7 domains: random sequence generation, allocation concealment, blinding of participants and investigators, the blindness of outcome assessments, incomplete outcome data, selective outcome reporting, and other biases.^[31]^ Each domain was classified as “high,” “low,” or “unclear” risk of bias. Any disagreement was rechecked by a discussion with a third reviewer (Huijuan Lou). Another 2 investigators (Huijuan Lou and Lili Chen) independently assessed the overall evidence and certainty of evidence for each outcome with the Grading of Recommendations Assessment, Development, and Evaluation (GRADE) framework, which divides evidence into very low, low, moderate, and high levels.^[32]^ The discrepancies were resolved by a third reviewer (Gaofeng Wang).

### 2.6. Data synthesis

The meta-analysis was performed using RevMan 5.4 provided by the Cochrane Collaboration.^[31]^ The mean difference (MD) with 95% confidence interval (CI) was calculated for each outcome before data pooling. Any heterogeneity among the included trials was evaluated by using *I*^2^ statistics. Specifically, 0 < *I*^2^ < 25% was defined as low heterogeneity, 25% < *I*^2^ < 50% as moderate heterogeneity, 50% < *I*^2^ < 75% as significant heterogeneity, and 75% < *I*^2^ < 100% as high heterogeneity.^[33–35]^ The random-effects model was applied for synthesizing data because of the diversity of massage forms across the included studies.^[36,37]^ The robustness of the pooled conclusion was assessed by using sensitivity analysis through the sequential exclusion of each trial. The *P*-value for the pooled conclusions was 2-sided, and the inspection level was 0.05. Potential publication bias was assessed by visualization of asymmetry in funnel plots (≥10 included studies).^[38]^ Preplanned subgroup analyses of our estimates of treatment effect were conducted to explore the influence of different ages (children and adolescents [<18 years] or adults [≥18 years]), different treatment intervals (continuous treatment or intermittent treatment), and different treatment times (treatment times ≤30 or treatment times >30).

## 3. Results

### 3.1. Literature search

**Figure 1.**
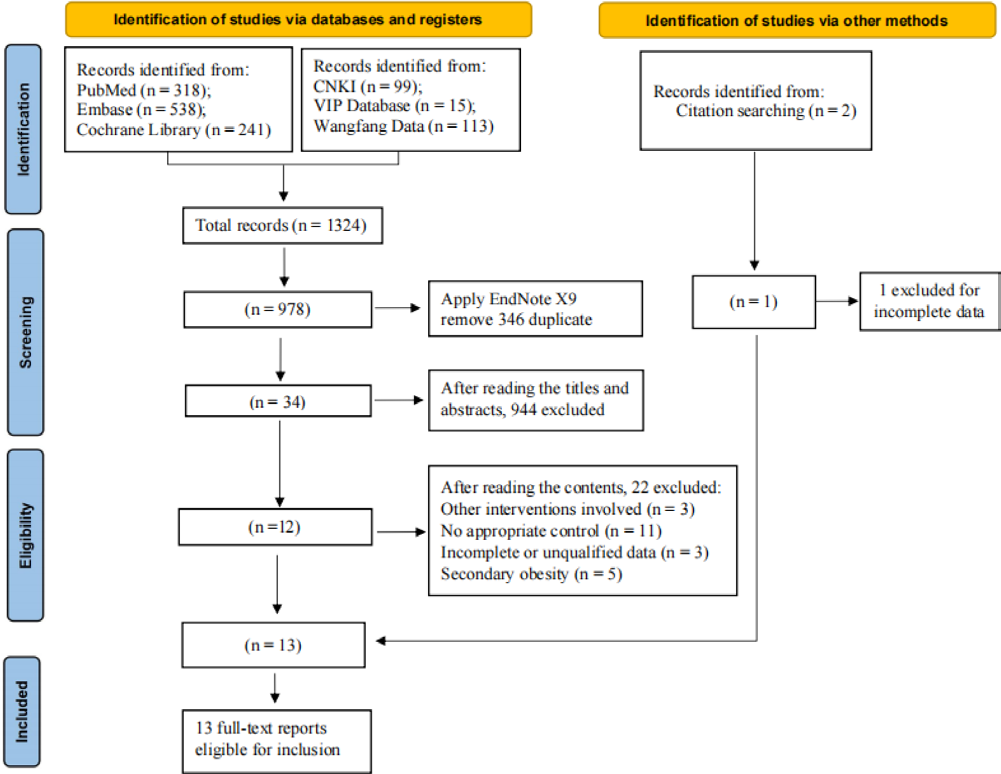
PRISMA flow chart for literature search and study selection. PRISMA, Preferred Reporting Items for Systematic Reviews and Meta-Analysis.

A total of 1324 articles were identified during the initial electronic search, and 978 articles were retained after removing 346 duplicate articles by EndNote X9. Then, 944 studies were excluded after reading the titles and abstracts, and the full-text versions of the remaining 34 articles were retrieved for further evaluation. A total of 21 studies were further excluded because of other interventions involved,^[39–41]^ lack of appropriate control,^[42–52]^ incomplete or unqualified data,^[53–55]^, and secondary obesity.^[56–60]^ One new eligible study^[61]^ was obtained by reviewing the reference lists of the included studies. Finally, a total of 13 studies were selected for the meta-analysis (Fig. 1).

### 3.2. Study characteristics and quality assessment

**Figure 2.**
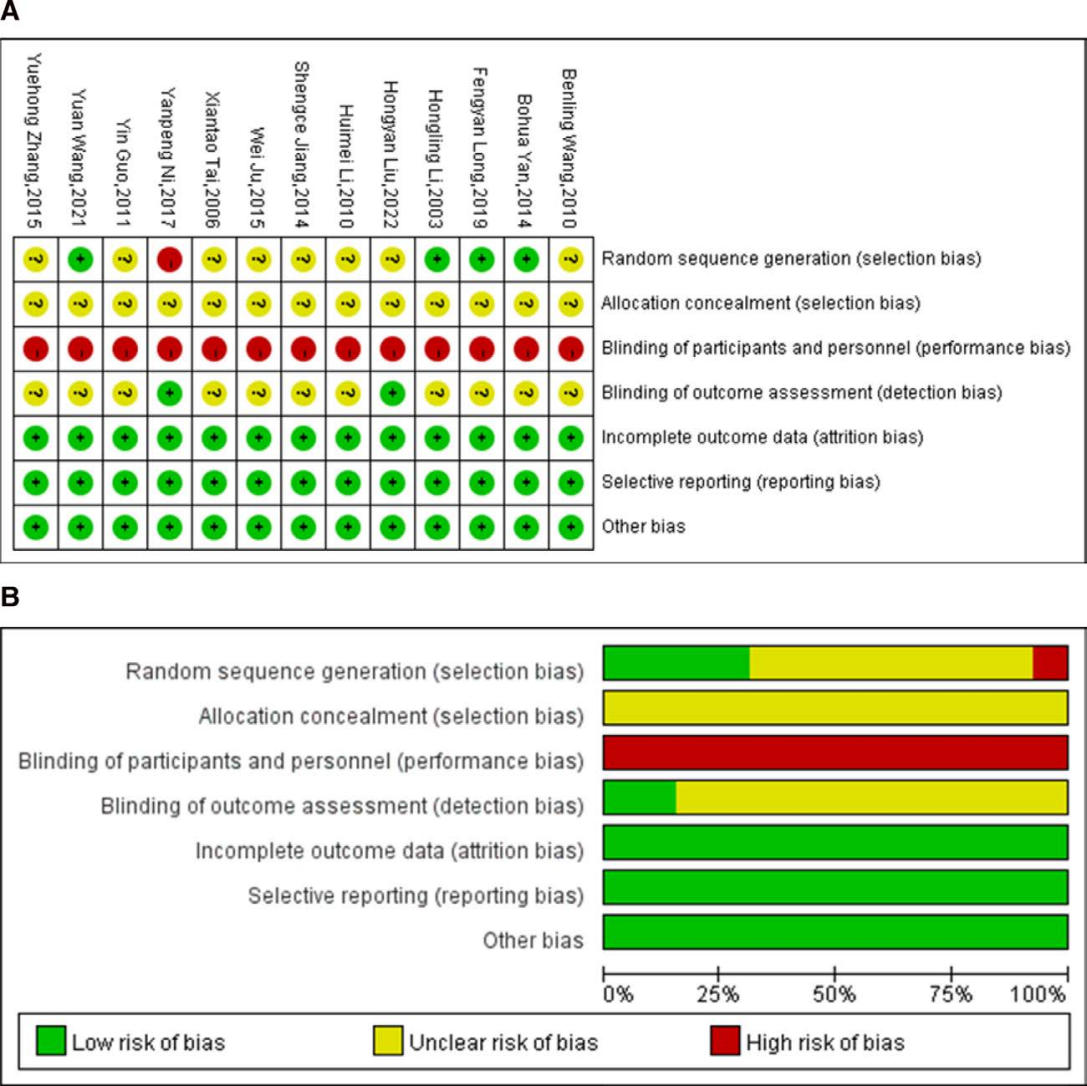
Risk of bias.

The baseline characteristics of the included studies are summarized in Table 2. All of the trials were carried out in China, and a total of 848 subjects with obesity were enrolled. The treatment duration ranged from 14 days to 5 months, and 18 to 116 individuals were included in each trial. The Cochrane RoB 2.0 tool was used to evaluate the methodological quality of each of the included RCTs (Fig. 2). There were some concerns related to bias associated with different reasons. Nine trials^[61–63,65,67–71]^ used a completely random design, with only 4 trials^[62,63,67,69]^ definitely describing computer-generated randomization. One trial^[66]^ grouped the participants based on their wishes. Three of the included studies^[64,72,73]^ failed to clearly describe random sequence generation. All the included trials were considered to have an unclear risk of bias for reporting no information about allocation concealment. Owing to the characteristics of massage, it is difficult to conduct blinding for participants and personnel; hence, all of the included studies^[61–73]^ were assessed as having a high risk of bias in the performance bias. None of the included studies^[61–73]^ reported their outcome-assessment methods. We judged all 13 trials^[61–73]^ as having a low risk of reporting bias given that the reported outcome measures were in accordance with those listed in the methods section. We did not identify any other potential sources of bias. In all, the overall bias of the 13 studies was mainly related to the allocation concealment, blinding of participants and investigators, and the blindness of outcome assessments.

**Table 2:** Characteristics of the included studies and patients.

Author (yr)	Location/language	Sample size (In: Co)	Age (yr)	Male (%)	Intervention	Control	Treatment duration	Inclusion criteria	Adverse events	Course of disease (yr)	Outcomes
Benling Wang (2010)^[59]^	Jinan, China/Chinese	60 (30/30)	18–65 yr	NA	D + E + M	D + E	8 wks	BMI ≥ 28.0 kg/m^2^	NA	NA	TC, TG, HDL-C, LDL-C
Bohua Yan (2014)^[61]^	Chengdu, China/English	60 (30/30)	30–55 yr/25–53 yr	10%	D + E + M	D + E	8 wks	BMI ≥ 25.0 kg/m^2^	NA	2.4–5.0 yr/2.0–7.0 yr	BW, BMI, WC, HC
Fengyan Long (2019)^[62]^	Fangchenggang, China/Chinese	95 (47/48)	10.03 ± 2.15 yr/9.82 ± 2.30 yr	57.90%	D + E + P + M	D + E + P	12 wks (84 d)	BMI > 26 kg/m^2^	None	2.73 ± 0.77 yr/2.75 ± 0.93 yr	BW, WC, BMI, TC, TG, HDL-C, LDL-C
Hongling Li (2003)^[63]^	Zhengzhou, China/Chinese	60 (30/30)	6–15 yr	80%	D + E + P + M	D + E + P	30 d	BMI > 30 kg/m^2^	NA	NA	BW, BMI
Hongyan Liu (2022)^[64]^	Beijing, China/Chinese	116 (58/58)	15.20 ± 2.15 yr/16.31 ± 2.09 yr	56.9%	E + M	E	4 wks (14 d)	BMI > 30 kg/m^2^	NA	NA	BW, BMI, TG, HDL-C, LDL-C, TC, 2hFPG.
Huimei Li (2010)^[65]^	Jinan, China/Chinese	67 (33/34)	19–64 yr/19–62 yr	29.90%	D + E + M	D + E	8 wks	BMI ≥ 28.0 kg/m^2^	NA	1–28 yr	BW, WC, HC, BMI
Shengce Jiang (2014)^[60]^	Duyun, China/Chinese	20 (10/10)	28.4 ± 3.9 yr/29.5 ± 5.4 yr	0%	D + E + P + M	D + E + P	12 wks (84 d)	BMI > 30 kg/m^2^	NA	NA	BW, BMI, TG, TC, LDL-C, HDL-C
Wei Ju (2015)^[66]^	Zhengzhou, China/Chinese	60 (30/30)	9–15 yr	NA	D + E + P + M	D + E + P	2 mo (60 d)	BMI > 30 kg/m^2^	NA	NA	BMI, TC, TG, LDL-C, HDL-C
Xiantao Tai (2006)^[67]^	Kunming, China/Chinese	38 (18/20)	6–14 yr	52.63%	D + E + P + M	D + E + P	15 wks (45 d)	BW > y × 2 + 8	Transient increased appetite	NA	BW, BMI; HC, WC, WHR
Yanpeng Ni (2017)^[68]^	Luoyang, China/Chinese	90 (45/45)	41.4 ± 7.5 yr/40.3 ± 6.7 yr	65.56%	D + E + M	D + E	5 mo	BMI > 24	NA	NA	BMI
Yin Guo (2011)^[69]^	Shanghai, China/Chinese	42 (24/18)	17.3 ± 3.6 yr/16.3 ± 2.8 yr	47.60%	D + E + P + M	D + E + P	4 wks (28 d)	BFI > 40%	NA	NA	BW, WC, HC, BMI
Yuan Wang (2021)^[70]^	Yaan, China/Chinese	80 (40/40)	6.34 ± 3.67 yr/6.88 ± 3.56 yr	52.50%	D + E + M	D + E	NA	BMI > 30 kg/m^2^	NA	NA	BW, BMI, WC, HC, FPG,2hPG, FINS, IAI, HOMA-IR
Yuehong Zhang (2015)^[71]^	Zhengzhou, China/Chinese	60 (30/30)	5–12 yr	65%	D + E + M	D + E	2 mo (60 d)	BMI > 30 kg/m^2^	NA	NA	BW, BMI, WC, HC, FPG, 2hPG, FINS, IAI, HOMA-IR

2hPG = 2-hour postprandial blood glucose, BMI = body mass index: body weight, Co = control, D = diet, E = exercise, FINS = fasting insulin, FPG = fasting plasma glucose, HC = hip circumference, HDL-C = high-density lipoprotein cholesterol, HOMA-IR = homeostasis model assessment–insulin resistance, IAI = insulin action index, In = intervention, LDL-C = low-density lipoprotein cholesterol, M = massage, P = psychology, TC = total cholesterol, TG = triglyceride, WC = waist circumference.

### 3.3. Primary outcomes

#### 3.3.1. Body weight

**Figure 3.**
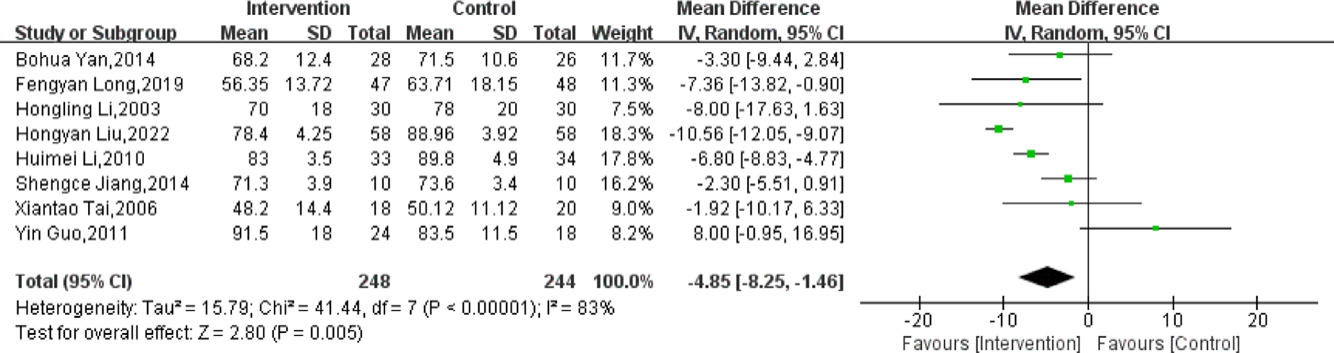
Effect of massage combined with lifestyle intervention compared with lifestyle intervention alone on body weight. CI= confidence interval, SD = standard deviation.

Eight trials^[62–65,68,69,71,73]^ reported the effect of massage combined with lifestyle intervention compared with lifestyle intervention alone on BW. There was a total of 492 subjects included, with 248 subjects in the experimental groups and 244 subjects in the control groups. There was significant heterogeneity across the included trials (*I*^2^ = 83%; *P* < .00001). Hence, the sources of heterogeneity were evaluated through sensitivity analysis. After deleting each single study one by one, there was no distinct change in the MD and 95% CI of the appraisal of BW, suggesting stable outcomes (Table S1, Supplemental Digital Content, http://links.lww.com/MD/O236). The outcomes showed that the use of massage combined with lifestyle intervention was associated with a decrease in BW compared with lifestyle intervention alone (MD: −4.85; 95% CI: −8.25 to −1.46; *P* = .005; Fig. 3). Based on the GRADE framework, the outcomes of massage combined with lifestyle intervention on BW compared with lifestyle intervention alone were considered as moderate-quality evidence (Table 3).

**Table 3:** GRADE evaluation and recommendations strength.

Outcomes	Relative effect (95% CI)	Importance	No. of participants (studies)	Quality of the evidence (GRADE)	Comments
BW	MD: −4.85 (−8.25 to −1.46)	Critical	492 (8 studies)	⊕⊕⊕⊝ Moderate^a^	May decrease the BW
BMI	MD: −2.65 (−4.05 to −1.24)	Critical	782 (12 studies)	⊕⊕⊝⊝ Low^a^^,^^b^	May decrease the BMI
WC	MD: −3.63 (−6.28 to −0.98)	Important	296 (5 studies)	⊕⊕⊝⊝ Low^a^^,^^c^	May lower the WC
HC	MD: −0.98 (−2.26 to 0.30)	Important	201 (4 studies)	⊕⊕⊝⊝ Low^a^^,^^c^	May lower the HC
TC	MD: −0.33 (−0.84 to 0.18)	Not important	351 (5 studies)	⊕⊝⊝⊝ Very low^a^^,^^b^^,^^c^	May lower the level of TC
TG	MD: −0.37 (−0.60 to −0.13)	Not important	351 (5 studies)	⊕⊕⊝⊝ Low^a^^,^^c^	May lower the level of TG
HDL-C	MD: −0.02 (−0.15 to 0.11)	Not important	351 (5 studies)	⊕⊕⊝⊝ Low^a^^,^^c^	No definite conclusion can be drawn
LDL-C	MD: −0.23 (−0.45 to −0.02)	Not important	351 (5 studies)	⊕⊕⊝⊝ Low^a^^,^^c^	May lower the level of HDL-C
FPG	MD: −0.10 (−0.22 to 0.02)	Not important	140 (2 studies)	⊕⊕⊝⊝ Low^a^^,^^c^	No definite conclusion can be drawn
2hPG	MD: 0.08 (−0.57 to 0.74)	Not important	140 (2 studies)	⊕⊕⊝⊝ Low^a^^,^^c^	No definite conclusion can be drawn
FINS	MD: −1.64 (−3.16 to −0.12)	Not important	140 (2 studies)	⊕⊕⊝⊝ Low^a^^,^^c^	May lower the level of FINS
HOMA-IR	MD: −0.42 (−0.65 to −0.18)	Not important	140 (2 studies)	⊕⊕⊝⊝ Low^a^^,^^c^	May lower the level of HOMA-IR
IAI	MD: −0.02 (−1.03 to 0.99)	Not important	140 (2 studies)	⊕⊝⊝⊝ Very low^a^^,^^b^^,^^c^	No definite conclusion can be drawn

2hPC = 2-hour postprandial blood glucose, BMI = body mass index, BW = body weight, CI = confidence interval, FINS = fasting insulin, FPG = fasting plasma glucose, HC = hip circumference, HDL-C = high-density lipoprotein cholesterol, HOMA-IR = homeostasis model assessment–insulin resistance, IAI = insulin action index, LDL-C = low-density lipoprotein cholesterol, MD = mean difference, TC = total cholesterol, TG = triglyceride, WC = waist circumference.

aDowngraded because of serious risk of bias (no blinding).

bDowngraded because of serious inconsistency (*I*^2^ = 90%, point estimates and CI vary considerably).

cDowngraded because of serious imprecision (small sample size).

d Downgraded because of serious inconsistency (*I*^2^ = 98%, point estimates and CI vary considerably).

#### 3.3.2. Body mass index

**Figure 4.**
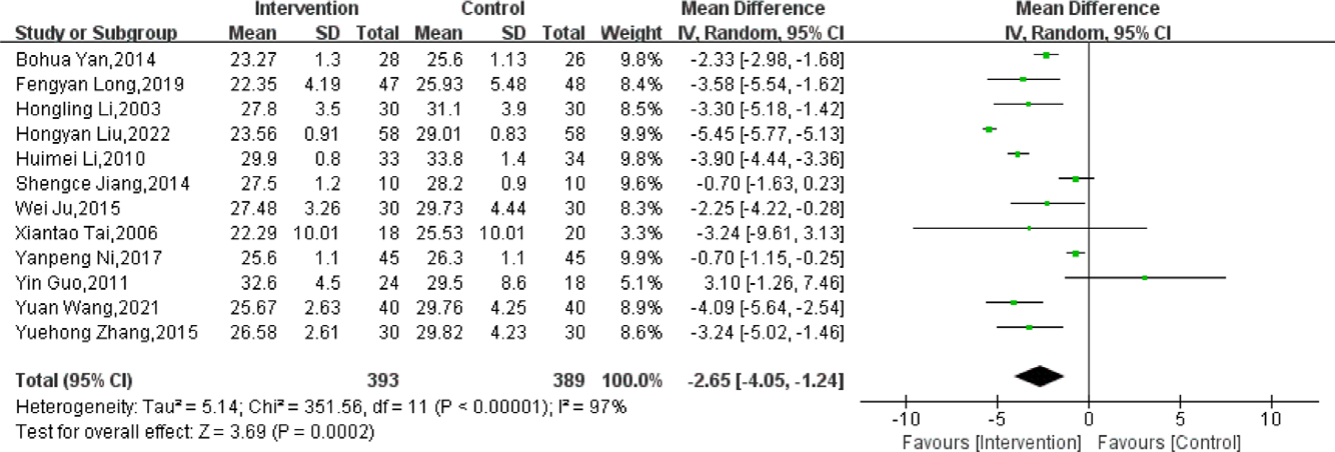
Effect of massage combined with lifestyle intervention compared with lifestyle intervention alone on body mass index. CI= confidence interval, SD = standard deviation.

Data on the effect of massage combined with lifestyle intervention compared with lifestyle intervention alone on BMI were reported in 12 trials.^[61–66,68–73]^ There was a total of 782 subjects included, with 393 subjects in the experimental groups and 389 subjects in the control groups. The results showed that there was a high heterogeneity between trials (*P* < .00001, *I*^2^ = 97%). As a result, the sources of heterogeneity were evaluated through sensitivity analysis. After excluding any single study one by one, no remarkable change in the MD and 95% CI of the evaluation of BMI was found, pointing out stable results (Table S2, Supplemental Digital Content, http://links.lww.com/MD/O236). In accordance with the results of the meta-analysis, massage combined with lifestyle intervention could decrease BMI (MD: −2.65; 95% CI: −4.05 to −1.24; *P* = .0002; Fig. 4) compared with lifestyle intervention alone. Based on the GRADE framework, the outcomes of BMI were classified as low-quality evidence (Table 3).

**Figure 5.**
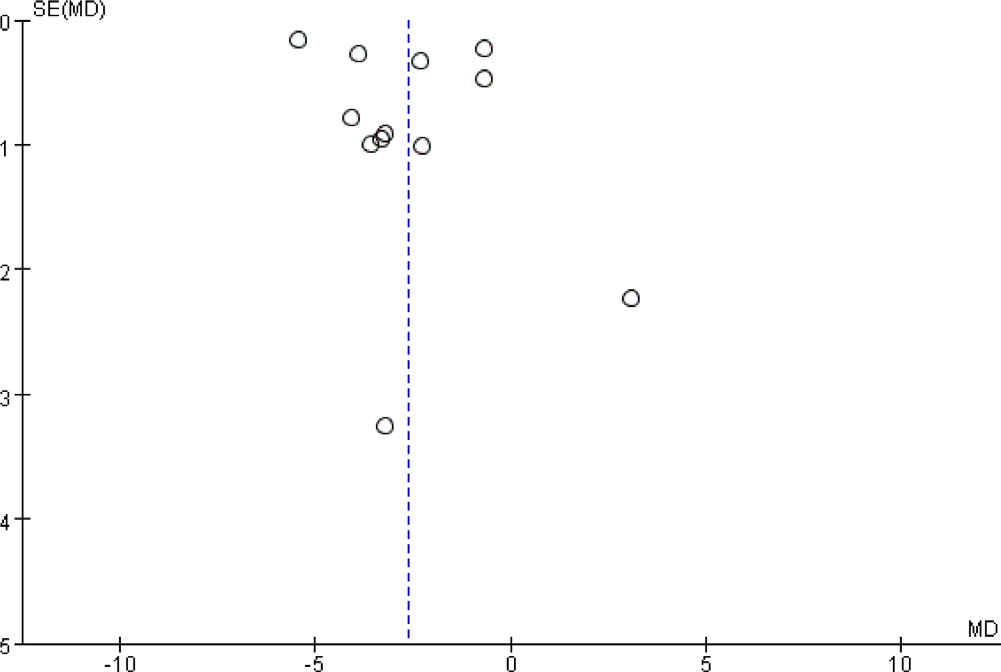
Funnel plot of the effect of massage combined with lifestyle intervention compared with lifestyle intervention alone on body mass index. MD = mean difference.

The number of included studies was >10, so the publication bias was examined. For all of the included studies involving BMI, the funnel plots showed no remarkable asymmetry, implying no obvious publication bias existed (Fig. 5).

### 3.4. The secondary outcomes

#### 3.4.1. Waist circumference and hip circumference

**Figure 6.**
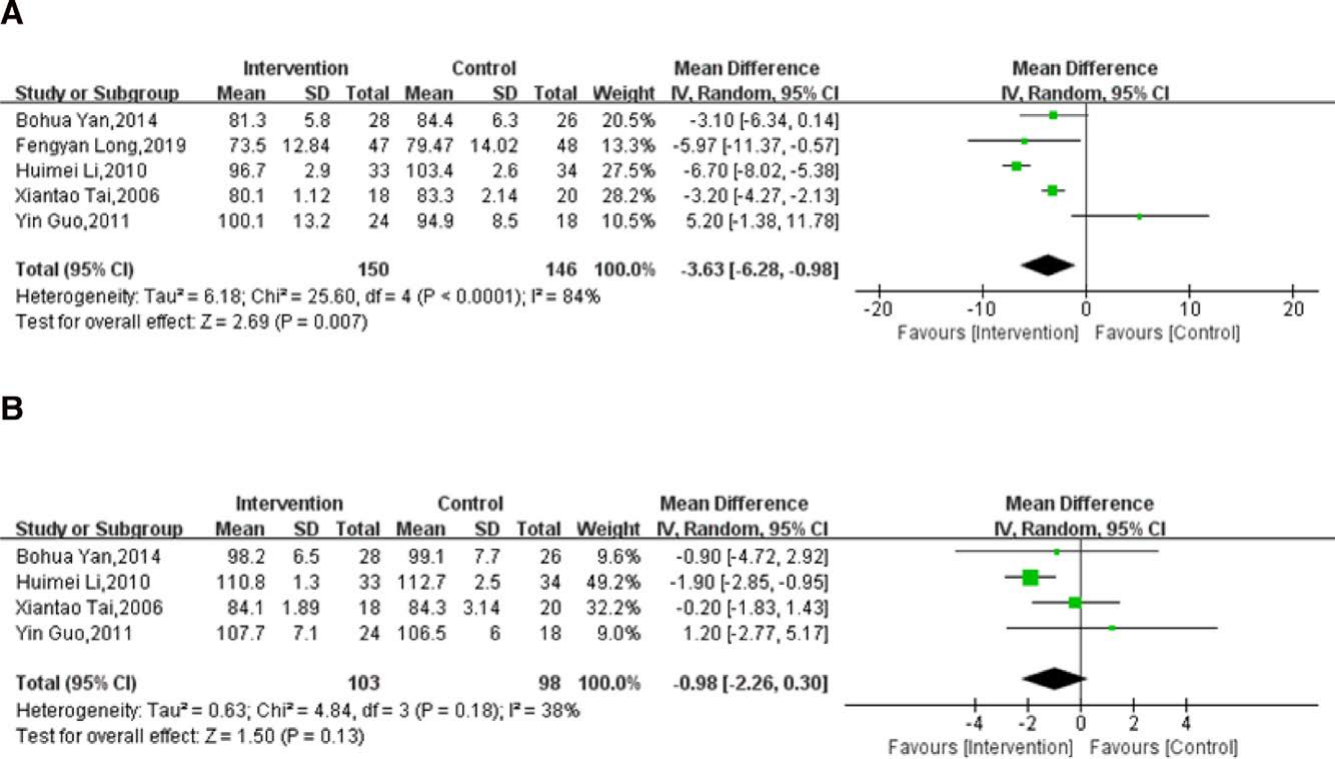
Effect of massage combined with lifestyle intervention compared with lifestyle intervention alone on waist circumference (A) and hip circumference (B). CI= confidence interval, SD = standard deviation.

Five trials reported the effect of massage combined with lifestyle intervention compared with lifestyle intervention alone on WC.^[62–65,68]^ There was a total of 296 subjects included, with 150 subjects in the experimental groups and 146 subjects in the control groups. There was a high heterogeneity across the included trials (*I*^2^ = 84%; *P* < .0001). Thus, the sources of heterogeneity were detected through sensitivity analysis. After taking away the included trials one by one, there were no obvious changes in the MD and 95% CI of the assessment of WC (Table S3, Supplemental Digital Content, http://links.lww.com/MD/O236). The overall outcomes showed that the use of massage combined with lifestyle intervention was associated with a decrease in WC compared with lifestyle intervention alone (MD: −3.63; 95% CI: −6.28 to −0.98; *P* = .007; Fig. 6A).

Four trials^[62,64,65,68]^ reported the effect of massage combined with lifestyle intervention compared with lifestyle intervention alone on HC. There was a total of 201 subjects included, with 103 subjects in the experimental groups and 98 subjects in the control groups. There was moderate heterogeneity across the included trials (*I*^2^ = 38%; *P* = .18). The overall results showed that patients treated with massage combined with lifestyle intervention had no obvious reduction in HC (MD: −0.98; 95% CI: −2.26 to 0.30; *P* = .13; Fig. 6B) compared with lifestyle intervention alone.

In accordance with the GRADE framework, both the quality of WC and that of HC were classified as low-quality evidence (Table 3).

#### 3.4.2. Serum lipids

Data on the effect of massage combined with lifestyle intervention compared with lifestyle intervention alone on 4 different blood serum lipid components (total cholesterol [TC], triglyceride [TG], low-density lipoprotein cholesterol [LDL-C], and high-density lipoprotein cholesterol [HDL-C]) were all reported in the same 5 trials.^[61,63,71–73]^ There was a total of 351 participants included, with 175 participants in the experimental groups and 176 participants in the control groups.

**Figure 7.**
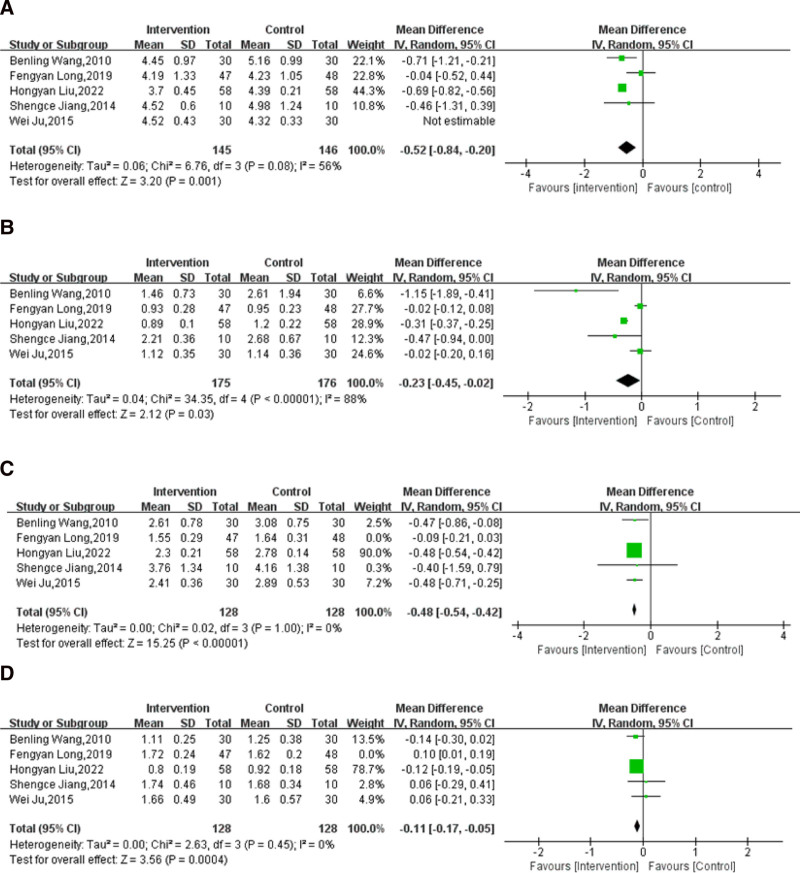
Effect of on massage combined with lifestyle intervention compared with lifestyle intervention on different blood serum lipid components: (A) TC, (B) TG, (C) LDL-C, and (D) HDL-C. CI = confidence interval, HDL-C = high-density lipoprotein cholesterol, LDL-C = low-density lipoprotein cholesterol, SD = standard deviation, TC = total cholesterol, TG = triglyceride.

For TC, a significant heterogeneity was observed among the included trials (*I*^2^ = 93%; *P* < .00001). To assess the sources of heterogeneity, the sensitivity analysis was conducted by removing the included studies one by one. After taking away the most heterogeneous study^[72]^ (Table S4, Supplemental Digital Content, http://links.lww.com/MD/O236), the overall results showed that the use of massage combined with lifestyle intervention was associated with an evident decrease in TC compared with lifestyle intervention alone (*P* = .08, *I*^2^ = 56%; MD: −0.52; 95% CI: −0.84 to −0.20; *P* = .001; Fig. 7A).

For TG, apparent heterogeneity was detected among the included studies (*I*^2^ = 88%; *P* < .00001). This result was not clearly altered after cutting out the individual trials one by one (Table S5, Supplemental Digital Content, http://links.lww.com/MD/O236). The ultimate outcomes showed that the application of massage combined with lifestyle intervention could reduce the level of TG compared with lifestyle intervention alone (MD: −0.23; 95% CI: −0.45 to −0.02; *P* = .003; Fig. 7B).

For LDL-C, distinct heterogeneity was detected among the included trials (*I*^2^ = 88%; *P* < .00001). The sources of heterogeneity were thereby appraised by sensitivity analysis. After removing the most heterogeneous study^[63]^ (Table S6, Supplemental Digital Content, http://links.lww.com/MD/O236), the results showed that the application of massage combined with lifestyle intervention was related to a decrease in LDL-C compared with lifestyle intervention alone (*P* = 1.00, *I*^2^ = 0%; MD: −0.48; 95% CI: −0.54 to −0.42; *P* < .00001; Fig. 7C).

In HDL-C level, heterogeneity was found among the included trials (*I*^2^ = 77%; *P* = .002). By removing the most heterogeneous study^[63]^ (Table S7, Supplemental Digital Content, http://links.lww.com/MD/O236), the overall results showed that the application of massage combined with lifestyle intervention was related to a decrease in the level of HDL-C compared with lifestyle intervention alone (*P* = .45, *I*^2^ = 0%; MD: −0.11; 95% CI: −0.17 to −0.05; *P* = .0004; Fig. 7D).

In accordance with the GRADE framework, the quality of evidence of TC, TG, LDL-C, and HDL-C outcomes was classified as very low, low, low, and low, respectively (Table 3).

#### 3.4.3. Blood glucose and insulin sensitivity

**Figure 8.**
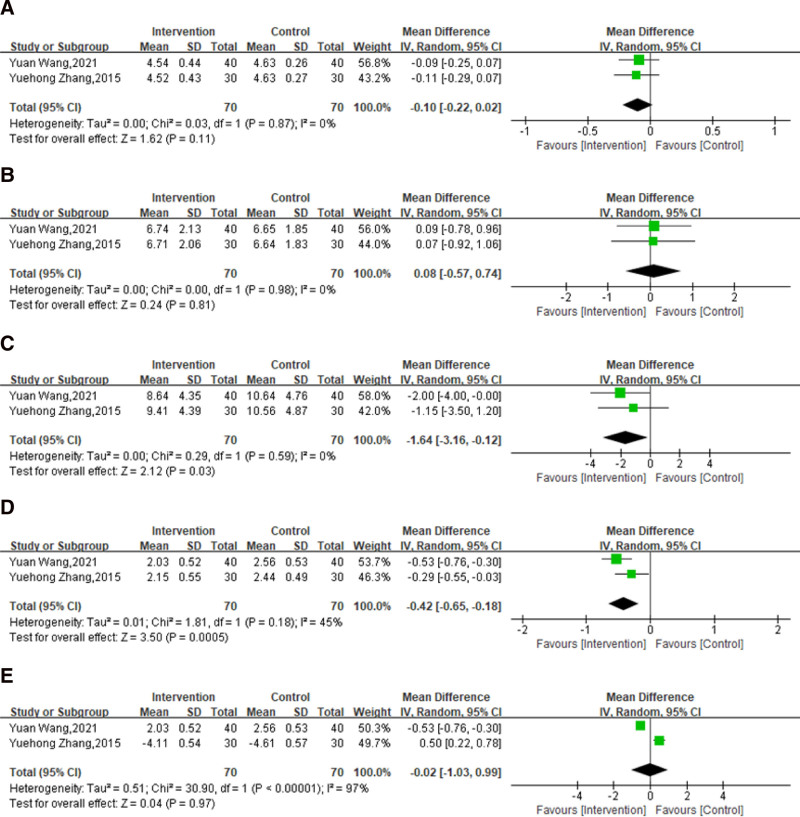
Effect of massage combined with lifestyle intervention compared with lifestyle intervention on blood glucose and insulin sensitivity: (A) FPG, (B) 2hPC, (C) FINS, (D) HOMA-IR, and (E) IAI. 2hPC, 2-hour postprandial blood glucose, CI= confidence interval, FINS = fasting insulin, FPG = fasting plasma glucose, HOMA-IR = homeostasis model assessment–insulin resistance, IAI = insulin action index, SD = standard deviation.

Data on the effect of massage combined with lifestyle intervention compared with lifestyle intervention alone on blood glucose and insulin sensitivity (fasting plasma glucose [FPG], 2-hour postprandial blood glucose, fasting insulin [FINS], homeostasis model assessment–insulin resistance [HOMA-IR], and insulin action index [IAI]) were reported in the same 2 trials.^[67,70]^ There was a total of 140 subjects included, with 70 subjects in the experimental groups and 70 subjects in the control groups. As shown in Figure 8, there was no significant change of FPG (*P* = .87, *I*^2^ = 0%; MD: −0.10; 95% CI: −0.22 to 0.02; *P* = .11; Fig. 8A), 2hPG (*P* = .98, *I*^2^ = 0%; MD: 0.08; 95% CI: −0.57 to 0.74; *P* = .81; Fig. 8B) and IAI (*P* < .00001, *I*^2^ = 97%; MD: −0.02; 95% CI: −1.03 to 0.99; *P* = .97; Fig. 8E) after massage combined with lifestyle intervention compared with lifestyle intervention alone. However, the results illustrated that the use of massage combined with lifestyle intervention was related to a decrease in FINS (*P* = .59, *I*^2^ = 0%; MD: −1.64; 95% CI: −3.16 to −0.12; *P* = .03; Fig. 8C) and HOMA-IR (*P* = .18, *I*^2^ = 45%, MD: −0.42; 95% CI: −0.65 to−0.18; *P* = .0005; Fig. 8D) compared with lifestyle intervention alone.

The quality of evidence for FPG, 2-hour postprandial blood glucose, FINS, and HOMA-IR was low, and that for IAI was very low in accordance with the GRADE framework (Table 3).

#### 3.4.4. Adverse events

Adverse events were reported in only 2 trials. One study reported no adverse events,^[63]^ and the other reported transiently increased appetite.^[68]^

### 3.5. Subgroup analyses

#### 3.5.1. Subgroup analyses by different ages

Since some of the included studies focused on children and adolescents (<18 years), while others focused on adult or elderly subjects (≥18 years), the effects may vary among different age groups. So, we carried out subgroup analyses after stratifying for the age of the subjects. The results revealed that no significant differences between subgroups were detected in BW (*P* = .97, *I*^2^ = 0%), WC (*P* = .27, *I*^2^ = 16.4%), TC (*P* = .26, *I*^2^ = 20.8%), TG (*P* = .62, *I*^2^ = 0%), LDL-C (*P* = .08, *I*^2^ = 67.9%), and HDL-C (*P* = .36, *I*^2^ = 0%), but more obvious reduction in BMI (*P* = .02, *I*^2^ = 80.3%) for the children and adolescents (<18 years) subgroup and more obvious reduction in HC (*P* = .04, *I*^2^ = 76.1%) for the adults (≥18 years) subgroup (Table 4).

**Table 4:** Subgroup analyses by different ages.

Outcomes	Children and adolescents subgroup		Adults subgroup		Subgroup differences
	MD (95% CI)	Heterogeneity	MD (95% CI)	Heterogeneity	
BW	−4.65 (−10.88, 1.58)	*P* = .0004, *I*^2^ = 80%	−4.50 (−7.89, −1.11)	*P* = .05, *I*^2^ = 66%	*P* = .97, *I*^2^ = 0%
BMI	−3.79 (−5.00, −2.58)	*P* = .0004, *I*^2^ = 76%	−1.49 (−3.08, 0.10)	*P* < .00001, *I*^2^ = 96%	*P* = .02, *I*^2^ = 80.3%
WC	−1.93 (−6.68, .81)	*P* = .03, *I*^2^ = 72%	−5.22 (−8.69, −1.74)	*P* = .04, *I*^2^ = 75%	*P* = .27, *I*^2^ = 16.4%
HC	0.00 (−1.51, 1.51)	*P* = .52, *I*^2^ = 0%	−1.84 (−2.76, −0.92)	*P* = .62, *I*^2^ = 0%	*P* = .04, *I*^2^ = 76.1%
TC	−0.19 (−0.87, 0.50)	*P* < .00001, *I*^2^ = 97%	−0.65 (−1.08, −0.22)	*P* = .62, *I*^2^ = 0%	*P* = .26, *I*^2^ = 20.8%
TG	−0.12 (−0.35, 0.10)	*P* < .00001, *I*^2^ = 93%	−0.75 (−1.40, −0.09)	*P* = .13, *I*^2^ = 57%	*P* = .08, *I*^2^ = 67.9%
LDL-C	−0.35 (−0.63, −0.06)	*P* < .00001, *I*^2^ = 94%	−0.46 (−0.83, −0.10)	*P* = .91, *I*^2^ = 0%	*P* = .62, *I*^2^ = 0%
HDL-C	0.00 (−0.17, 0.18)	*P* = .0004, *I*^2^ = 87%	−0.10 (−0.25, 0.05)	*P* = .31, *I*^2^ = 1%	*P* = .36, *I*^2^ = 0%

BMI = body mass index, BW = body weight, HC = hip circumference, HDL-C = high-density lipoprotein cholesterol, LDL-C = low-density lipoprotein cholesterol, TC = total cholesterol, TG = triglyceride, WC = waist circumference.

#### 3.5.2. Subgroup analyses by different treatment intervals

Given that the massage treatment interval varied among the included studies, including once a day, once every other day, or 2 to 3 times a week with a rest at the weekend, subgroup analyses were conducted according to different treatment intervals. We define daily massage as continuous treatment, while treatment once every other day or 2 to 3 times a week with weekend rest as intermittent treatment. The results revealed that no obvious differences between subgroups were presented in BW (*P* = .20, *I*^2^ = 39.2%), BMI (*P* = .45, *I*^2^ = 0%), WC (*P* = .16, *I*^2^ = 48.6%), HC (*P* = .25, *I*^2^ = 23.5%), and TG (*P* = .16, *I*^2^ = 49.5%), but more significant reduction in TC (*P* < .00001, *I*^2^ = 98.3%), LDL-C (*P* < .00001, *I*^2^ = 95.6%), and HDL-C (*P* < .0001, *I*^2^ = 94.1%) for intermittent treatment subgroup (Table 5).

**Table 5:** Subgroup analyses by different ages.

Outcomes	Continuous treatment		Intermittent treatment		Subgroup differences
	MD (95% CI)	Heterogeneity	MD (95% CI)	Heterogeneity	
BW	−2.63 (−8.23, 2.98)	*P* = .03, *I*^2^ = 66%	−6.95 (−10.43, −3.46)	*P* = .002, *I*^2^ = 80%	*P* = .20, *I*^2^ = 39.2%
BMI	−2.08 (−3.53, −0.64)	*P* = .002, *I*^2^ = 73%	−3.11 (−5.35, −0.87)	*P* < .00001, *I*^2^ = 99%	*P* = .45, *I*^2^ = 0%
WC	−1.47 (−5.65, 2.71)	*P* = .01, *I*^2^ = 85%	−4.50 (−5.30, −3.69)	*P* = .0002, *I*^2^ = 88%	*P* = .16, *I*^2^ = 48.6%
HC	1.20 (−2.77, 5.17)	Not applicable	−1.22 (−2.46, 0.01)	*P* = .20, *I*^2^ = 38%	*P* = .25, *I*^2^ = 23.5%
TC	0.14 (−0.04, 0.32)	*P* = .25, *I*^2^ = 29%	−0.69 (−0.82, −0.57)	*P* = .94, *I*^2^ = 0%	*P* < .00001, *I*^2^ = 98.3%
TG	−0.06 (−0.20, 0.09)	*P* = .18, *I*^2^ = 41%	−0.65 (−1.45, 0.16)	*P* = .03, *I*^2^ = 80%	*P* = .16, *I*^2^ = 49.5%
LDL-C	−0.18 (−0.28, −0.07)	*P* = .01, *I*^2^ = 77%	−0.48 (−0.54, −0.42)	*P* = .96, *I*^2^ = 0%	*P* < .00001, *I*^2^ = 95.6%
HDL-C	0.09 (0.01, 0.18)	*P* = .94, *I*^2^ = 0%	−0.12 (−0.19, −0.06)	*P* = .82, *I*^2^ = 0%	*P* < .0001, *I*^2^ = 94.1%

BMI = body mass index, BW = body weight, HC = hip circumference, HDL-C = high-density lipoprotein cholesterol, LDL-C = low-density lipoprotein cholesterol, TC = total cholesterol, TG = triglyceride, WC = waist circumference.

#### 3.5.3. Subgroup analyses by different treatment times

The number of treatments for subjects varies among the included studies, with 6 studies receiving less than or equal to 30 treatments, 6 studies conducting more than 30 treatments, and 1 study not clearly describing the number of treatments. Subgroup analyses were conducted by different treatment times (treatment times ≤30, treatment times >30). The results showed that no significant differences between subgroups were found in BW (*P* = .34, *I*^2^ = 0%), BMI (*P* = .16, *I*^2^ = 48.9%), WC (*P* = .76, *I*^2^ = 0%), HC (*P* = .26, *I*^2^ = 22.0%), TG (*P* = .16, *I*^2^ = 49.5%), LDL-C (*P* = .26, *I*^2^ = 20.9%), but more significant reduction in TC (*P* < .00001, *I*^2^ = 95.9%) and HDL-C (*P* < .0001, *I*^2^ = 94.1%) for treatment times ≤30 subgroup (Table 6).

**Table 6:** Subgroup analyses by different treatment times.

Outcomes	Treatment times ≤ 30		Treatment times > 30		Subgroup differences
	MD (95% CI)	Heterogeneity	MD (95% CI)	Heterogeneity	
BW	−5.55 (−9.68, −1.42)	*P* < .0001, *I*^2^ = 84%	−3.15 (−5.86, −0.44)	*P* = .37, *I*^2^ = 0%	*P* = .34, *I*^2^ = 0%
BMI	−3.18 (−4.79, −1.58)	*P* < .00001, *I*^2^ = 96%	−1.82 (−2.86, −0.77)	*P* = .005, *I*^2^ = 70%	*P* = .16, *I*^2^ = 48.9%
WC	−2.49 (−7.66, 2.67)	*P* = .0005, *I*^2^ = 87%	−3.30 (−4.36, −2.25)	*P* = .32, *I*^2^ = 0%	*P* = .76, *I*^2^ = 0%
HC	−1.44 (−2.82, −0.05)	*P* = .30, *I*^2^ = 16%	−0.20 (−1.83, 1.43)	Not applicable	*P* = .26, *I*^2^ = 22.0%
TC	−0.69 (−0.82, −0.57)	*P* = .94, *I*^2^ = 0%	0.08 (−0.20, 0.36)	*P* = .25, *I*^2^ = 29%	*P* < .00001, *I*^2^ = 95.9%
TG	−0.65 (−1.45, 0.16)	*P* = .03, *I*^2^ = 80%	−0.06 (−0.20, 0.09)	*P* = .18, *I*^2^ = 41%	*P* = .16, *I*^2^ = 49.5%
LDL-C	−0.48 (−0.54, −0.42)	*P* = .96, *I*^2^ = 0%	−0.28 (−0.62, 0.06)	*P* = .01, *I*^2^ = 77%	*P* = .26, *I*^2^ = 20.9%
HDL-C	−0.12 (−0.19, −0.06)	*P* = .82, *I*^2^ = 0%	0.09 (0.01, 0.18)	*P* = .94, *I*^2^ = 0%	*P* < .0001, *I*^2^ = 94.1%

BMI = body mass index, BW = body weight, HC = hip circumference, HDL-C = high-density lipoprotein cholesterol, LDL-C = low-density lipoprotein cholesterol, TC = total cholesterol, TG = triglyceride, WC = waist circumference.

## 4. Discussion

### 4.1. Principal findings

The systematic review and meta-analysis evaluated the comparative effectiveness of massage combined with lifestyle intervention and lifestyle intervention alone for simple obesity. A total of 848 subjects with simple obesity from 13 RCTs were included. The use of massage combined with lifestyle intervention significantly decreased BW, BMI, WC, TC, TG, LDL-C, FINS, HOMA-IR, but had less effect in increasing HDL-C, compared with lifestyle intervention alone.

BMI is the cornerstone of the current classification system for obesity,^[74]^ but it is actually a measure of height/weight relationships and tells us nothing about the underlying heterogeneity of obesity.^[75]^ While WC is an effective surrogate measure for central or visceral adipose tissue in comparison with BMI, which is an effective measure of total adiposity.^[76]^ HC is a more specific measure of subcutaneous adipose tissue.^[76]^ From this meta-analysis, we found that massage combined with lifestyle intervention not only has a stronger effect in reducing BW and BMI but also has a stronger effect in reducing WC compared with lifestyle intervention alone, which means massage combined with lifestyle intervention is more effective in reducing visceral adipose tissue compared with lifestyle intervention alone.

Usually, obesity is intimately associated with dyslipidemia.^[77]^ Elevated TG, and moderately elevated LDL-C accompanied by decreased HDL-C are the main characteristics of dyslipidemia caused by obesity.^[78]^ In this study, massage combined with lifestyle intervention resulted in more decline on the level of TC, TG, and LDL-C compared with lifestyle intervention alone. Therefore, massage combined with lifestyle intervention has a stronger ability to improve dyslipidemia compared with lifestyle intervention alone. Interestingly, massage combined with lifestyle intervention is not as effective as lifestyle intervention alone in increasing HDL-C, which is worth further exploration.

Overweight/obese is usually associated with enhanced insulin resistance^[79]^ and a heightened risk of diabetes, cardiovascular diseases, and premature mortality.^[76,80]^ Insulin resistance is the main driving force behind the development of dyslipidemia.^[77]^ In this study, compared with lifestyle intervention alone, massage combined with lifestyle intervention also produced more decline on the level of FINS and HOMA-IR, indicating more improvement in insulin resistance.

So, the results of this study showed that massage combined with lifestyle intervention can further improve the effect of lifestyle intervention on obesity. Therefore, in clinical practice, massage can be used in conjunction with lifestyle interventions to manage obesity. As research deepens, massage therapy may be widely used as an evidence-based complementary and alternative therapy for weight loss treatment.

### 4.2. Implications from subgroup analyses

In subgroup analyses by different ages, more obvious reduction in BMI occurred in the children and adolescents (<18 years) subgroup but more obvious reduction in HC presented in the adults (≥18 years) subgroup. The underlying reason is worth further exploration in the future.

In subgroup analyses by different treatment intervals, more significant reduction was detected in TC, LDL-C, and HDL-C for intermittent treatment subgroup, suggesting a better ability in reducing the levels of TC and LDL-C, but a slightly inferior ability in improving the HDL-C level for intermittent massage therapy compared with continuous massage therapy. That is to say, the effect of continuous massage intervention may not necessarily be better than intermittent massage intervention. Therefore, the optimal treatment interval also needs to be carefully considered in the future.

In subgroup analyses by different treatment times, no significant differences between subgroups were found in BW, BMI, WC, HC, TG, and LDL-C, meaning no sustained improvement in effectiveness as the course of treatment prolongs. However, more significant reduction in TC and less increase in HDL-C occurred in treatment times ≤30 subgroup compared with treatment times >30 subgroup, so, is the effect of massage on blood lipids temporary or has other reasons, which is worth exploring further.

### 4.3. Strengths of this study

There are 2 obvious strengths to our study. First, this review provides the most comprehensive synthesis of the evidence on massage for weight loss to date. Different from the previous review, we only included RCTs comparing the effectiveness of massage combined with lifestyle intervention and lifestyle intervention alone for simple obesity, making this review the most comprehensive update on the evidence of massage for simple obesity. Second, subgroup analyses were conducted by different ages, different treatment intervals and different treatment times, identifying some detailed issues in clinical practice.

### 4.4. Limitations of this study

This study has some limitations that should be addressed in future studies. First, among the included trials, a high risk of bias existed because of the lack of sufficient level of blinding. In the included trials, only 4 trials definitely described computer-generated randomization, but none of the 13 trials mentioned the method of blinding. In this review, substantial heterogeneity was observed and contributed to lowering the evidence grade from moderate to very low. Although blinding of subjects and investigators in massage and exercise intervention studies would be difficult, blinding of subjects and outcome assessment should be carried out to minimize the performance and evaluation bias of the studies. Thus, more trials with higher methodology standards should be conducted in the future.

Second, the number of included trials and subjects was small, which may limit statistical power. To obtain more statistically significant results, large samples and multicenter randomized trials should be carried out in days to come.

Third, the duration of massage differed considerably among the different trials. The duration of treatment ranged from 4 weeks to 5 months, and the duration of each intervention ranged from 20 to 60 minutes. The frequency of massage also varied among the studies, including once a day, once every other day, or 2 to 3 times a week with a rest at the weekend. Exercise therapy was also diversified. Despite the subgroup analysis was conducted by different treatment intervals and different treatment times in our meta-analysis, it is difficult to introduce concrete suggestions on the frequency and time of intervention, and the optimal treatment interval and the most appropriate treatment session length still need to be carefully considered in the future.

Fourth, the manipulation and acupoints differed across the included studies, with no unified manipulation and acupoint selection.

Fifth, in this meta-analysis, the language types of literature are limited in Chinese and English with no access to other important foreign language databases, and all of the studies had been conducted in China. In addition, most funnel plots were not feasible owing to the limited number of trials included for each comparison.

Sixth, none of the included trials reported the compliance rate. Low compliance rate is a disadvantage of lifestyle intervention for obesity. If massage combined with lifestyle intervention could improve compliance compared with lifestyle intervention alone, there would be strong evidence to support massage as a means to help lose weight.

Seventh, only 1^[69]^ of the 13 trials reported the follow-up after treatment. The long-term efficacy of massage combined with lifestyle intervention on obesity deserves further attention.

Finally, only 2^[63,68]^ of the 13 trials described adverse events. Understanding the adverse events associated with massage combined with lifestyle intervention for weight loss can better guide clinical practice. Therefore, continuous reporting of adverse events is necessary.

## 5. Conclusions

The systematic review and meta-analysis showed that the use of massage combined with lifestyle intervention significantly decreased BW, BMI, WC, TC, TG, LDL-C, FINS, and HOMA-IR compared with lifestyle intervention alone, and massage combined with lifestyle intervention is not as effective as lifestyle intervention alone in increasing HDL-C. Subgroup analyses presented age, treatment intervals, and treatment times may also affect treatment outcomes. And, our results highlight the need for research with high quality of trial and high certainty of evidence, and for studies considering different ages, different treatment intervals, and different treatment times.

## Acknowledgments

We thank all the authors for their contributions to this systematic review.

## Author contributions

**Data curation:** Gaofeng Wang, Lili Zhang, Lili Chen, Huijuan Lou.

**Formal analysis:** Gaofeng Wang, Lili Zhang, Lili Chen, Huijuan Lou.

**Writing – original draft:** Gaofeng Wang, Hongyu Ju.

**Conceptualization:** Hongyu Ju, Zepeng Zhang, Xingquan Wu, Heli Niu, Yonggang Yang.

**Methodology:** Zepeng Zhang, Xingquan Wu, Heli Niu, Yonggang Yang.

**Writing – review & editing:** Zepeng Zhang, Xingquan Wu, Heli Niu, Yonggang Yang.

**Supervision:** Yonggang Yang.
